# Erythrocyte fatty acid profiles in children are not predictive of autism spectrum disorder status: a case control study

**DOI:** 10.1186/s40364-018-0125-z

**Published:** 2018-03-14

**Authors:** Daniel P. Howsmon, James B. Adams, Uwe Kruger, Elizabeth Geis, Eva Gehn, Juergen Hahn

**Affiliations:** 1 Department of Chemical & Biological Engineering, Rensselaer Polytechnic Institute, 110 Eighth Street, Troy, 12180 NY USA; 2 Center for Biotechnology and Interdisciplinary Studies, Rensselaer Polytechnic Institute, 110 Eighth Street, Troy, 12180 NY USA; 3 School for Engineering of Matter, Transport, and Energy, Arizona State University, PO Box: 876106, Tempe, 85281 AZ USA; 4 Department of Biomedical Engineering, Rensselaer Polytechnic Institute, 110 Eighth Street, Troy, 12180 NY USA; 5 Willerson Center for Cardiovascular Modeling and Simulation, Institute for Computational Engineering and Sciences, University of Texas at Austin, Austin, 78712 TX USA

**Keywords:** Autism spectrum disorder, Fatty acids, Diagnostic biomarkers, Multivariate statistical analysis

## Abstract

Biomarkers promise biomolecular explanations as well as reliable diagnostics, stratification, and treatment strategies that have the potential to help mitigate the effects of disorders. While no reliable biomarker has yet been found for autism spectrum disorder (ASD), fatty acids have been investigated as potential biomarkers because of their association with brain development and neural functions. However, the ability of fatty acids to classify individuals with ASD from age/gender-matched neurotypical (NEU) peers has largely been ignored in favor of investigating population-level differences. Contrary to existing work, this classification task between ASD and NEU cohorts is the main focus of this work. The data presented herein suggest that fatty acids do not allow for classification at the individual level.

**Electronic supplementary material:**

The online version of this article (10.1186/s40364-018-0125-z) contains supplementary material, which is available to authorized users.

## Background

Autism spectrum disorder (ASD) comprises a broad class of psychological disorders characterized by compromised social communication/interaction and the presence of restricted, repetitive patterns of behavior [1]. The prevalence of ASD has increased markedly from 0.64% in 2002 to 1.14% in 2008 [2], a rate which exceeds that of other developmental disabilities [3]. Despite the high prevalence rates, the impaired quality of life associated with ASD [4], and substantial health care costs to families [5], the biochemical basis for ASD is largely unknown and therefore still an active area of research. Currently, ASD is only diagnosed and assessed through a variety of psychometric tools. However, numerous research efforts investigating potential biomarkers of and therapeutic strategies for ASD are ongoing.

Post-mortem brain analysis has revealed several structural and functional abnormalities associated with ASD, including altered synapse connectivity/plasticity [6], decreased neuron size and increased neuron density in the amygdala and hippocampus [7], decreased Purkinje cell size and number in the cerebellum [7], neuroinflammation [8], and aberrant activity-dependent transcription/translation [8]. On the molecular scale, alterations in Wnt/ *β*-catenin signaling (corroborated by putative mechanisms for valproate-inducing and folate-protective contributions to ASD), Ca^2+^ signaling, and glutamatergic/GABAergic signaling have been implicated in ASD. It is this role in neuroplasticity, neurogenesis, and synaptogenesis [9] that have led to investigations of polyunsaturated fatty acids (PUFAs) as potential targets for biomarker development and therapeutic intervention in ASD. PUFAs are essential fatty acids: precursors *α*-linolenic acid (ALA, 18:3n-3) and linoleic acid (LA, 18:2n-6) must be obtained from the diet. The downstream products docosahexaenoic acid (DHA; 22:6n-3) and arachidonic acid (AA; 20:4n-6) are the most abundant PUFAs in the brain and are vital components of neuronal phospholipids.

Plasma and erythrocyte levels of DHA and other fatty acids have been shown to be moderately correlated with fatty acid concentrations in the brain [10]; thus, plasma and erythrocyte fatty acid profiles have been investigated as potential biomarkers for ASD. Table 1 summarizes the recent literature evidence for ASD-related differences in erythrocyte-membrane/plasma fatty acid profiles and a recent meta-analysis by Mazahery et al. [11] suggests that individuals with ASD have lower AA, DHA, and EPA than their neurotypical (NEU) peers. It is important to note that some researchers choose to represent their results in terms of absolute fatty acid concentration in the sample, whereas other researchers represent their results in terms of relative concentration (to reflect concentration in the erythrocyte membrane rather than the blood sample) and that these disparate methods of reporting results can alter conclusions [12].

**Table 1:** Fatty acids from erythrocyte membranes measured in this work

Reference	Number of subjects		Age/gender matched?	Age	Abs./Rel. FA Conc.	Differences reported (ASD versus control)							
	ASD	Control				n-6	n-3	AA	DHA	EPA	AA/DHA	AA/EPA	Other
Vancassel et al. 2001 [36]	15	18^a^	N/N	ASD: 3-17 DD: 1-19	Relative	N.S.	*↓*	N.S.	*↓*				*↓* n-3/n-6
Meguid et al. 2008 [37]	30	30	Y/Y	3-11	Absolute			*↓*	*↓*		*↑*		*↓* linolenic and linoleic acids
Pastural et al. 2009[38]	15	11^b^	N/N	ASD: 8.7 ±3.9 DD: 7.9 ±2.9	Relative			*↑*	*↑*	*↑*			
Bell et al. 2010 [15]	45	45	Y/Y	ASD: 7.5 ±3.5 DD: 7.5 ±3.6	Relative	N.S.	N.S.	N.S.	N.S.	N.S.		*↑*	*↓* ALA
Bell et al. 2010 [15]	38	38^a^	Y/Y	ASD: 7.5 ±3.5 DD: 6.0 ±3.3	Relative	*↓*	N.S.	N.S.	N.S.	N.S.		N.S.	
El-Ansary et al. 2011 [13]	26	26	Y/Y	ASD: 4-12 DD: 4-11	Absolute			*↓*	*↓*	N.S.			
Brigandi et al. 2015 [14]	121	110	N/N	3-17	Relative	*↓*	*↓*	*↓*	*↓*	N.S.			
Yui et al. 2016 [24]	30	20	Y/Y	ASD: 13.6 ±4.3 DD: 13.2 ±5.4	Absolute			*↓*	N.S.	*↑*	*↓*	*↓*	
Jory et al. 2016 [39]	11	15	Y/N	ASD: 3.1 ±0.8 DD: 3.9 ±1.1	Relative			*↓*	*↓*	*↓*	N.S.	N.S.	*↓* n-3/n-6 and linoleic acid
Parletta et al. 2016 [16]	85	79	N/Y	ASD: 5.3 ±2.1 DD: 8.3 ±2.5	Relative			*↓*	*↓*	*↓*		*↑*	*↓* n-3/n-6

^a^Control group was Developmentally Delayed instead of NEU

^b^Many of the NEU controls were siblings of the ASD group

A successful biomarker or therapeutic target for ASD requires the metabolite or metabolite panel to separate individuals with ASD from NEU controls and/or strongly correlate with ASD severity. Therefore, this ability to separate individuals with ASD and NEU participants is not appropriately assessed with hypothesis testing on population means. More appropriate metrics are given in terms of classification performance on individuals (e.g. sensitivity/specificity, C-statistic, etc.). El-Ansary et al. [13] reported their results in terms of sensitivity/specificity and ROC curves; however, they had limited sample sizes of 26 ASD and 26 NEU participants and they assessed participants on the basis of absolute erythrocyte concentrations. Furthermore, their observed near-perfect separation in multiple fatty acid measurements (e.g., C-statistic of 1.00 for AA) has not been observed in larger cohorts (e.g., AA from [14]).

The aim of this study was to compare the level of erythrocyte-membrane fatty acids in a large cohort of ASD and NEU participants, and assess the ability of multivariate classification to separate ASD and NEU participants. The results presented herein contrast many of the conclusions about fatty acid biomarkers for ASD in the scientific literature, even though (as it will be shown) some other reports can be interpreted differently if these biomarkers are assessed on the individual level rather than comparing population means/medians. While no conclusions about the effectiveness of treatments that seek to raise fatty acid concentrations can be drawn from this work, the results indicate that fatty acid measurements are not a viable biomarker for ASD classification.

## Methods

### Study population

This paper analyzes baseline (prior to treatment) data from a 12-month nutrition/dietary treatment study known as the ASU Comprehensive Nutrition/Diet Treatment Study. Erythrocyte fatty acid measurements were available for 63 ASD and 49 NEU participants with a median (IQR) age of 9.7 (6.7) years and 10.0 (6.3) years, respectively. The average effect size *d* (i.e., Cohen’s d) for the fatty acid measurements under investigation was estimated a priori to be between 0.18 and 2.4 using data from the three largest studies in Table 1 [14–16]. With a *d*,*α*, and *β* of 0.5, 0.1, and 0.8, respectively, the minimum sample size is calculated to be 49 samples per group. The sample size used in this work is also greater than 8 of 10 studies reported in Table 1 that found statistically significant differences between ASD and NEU populations. This study was approved by the Institutional Review Board of Arizona State University. Eligibility and exclusion criteria, characteristics of the study population (including comorbidities), and descriptions of autism severity and overall functioning assessments are presented in [17]. It is important to note that both ASD and NEU participants were not allowed to have taken nutritional supplements or restricted to abnormal diets in the previous two months to be eligible for this study. Furthermore, since seafood consumption is the largest contributor of n-3 fatty acids in the Western diet, parents/caregivers were required to report the number of seafood servings eaten by the participant per month. All data used in this study are provided in Additional file 1.

### Fatty acid measurements

Fatty acid measurements were measured by Doctor’s Data, a commercial laboratory approved by the Clinical Laboratory Improvement Amendments (CLIA) program operated by the US Department of Health and Human Services. Red blood cell fatty acids were quantified by a flame ionization detector. Red blood cells were washed and derivatized to their methyl esters and fatty acids were extracted according to carbon number. All fatty acid measurements are normalized by the concentration of total fatty acids in the sample. Table 2 defines notation for the fatty acids measured herein.

**Table 2:** Fatty acids from erythrocyte membranes measured in this work

Name	Abbreviation	C:D	Group
Arachidonic acid	AA	20:4	n-6
Dihomo- *γ*-linoleic acid	DGLA	20:3	n-6
Docosahexanoic acid	DHA	22:6	n-3
Eicosapentaenoic acid	EPA	20:5	n-3
Elaidic acid		18:1	n-9 (trans)
Linoleic acid	LA	18:2	n-6
Oleic acid		18:1	n-9
Palmitelaidic acid		16:1	n-9 (trans)
Palmitic acid		16:0	—
Palmitoleic acid		17:1	n-7
Stearic acid		18:0	—

### Statistical analysis

#### Hypothesis testing

Individual measurements for each cohort were first assessed for normality with the Anderson-Darling test [18] at a significant level of 0.05. If distributions from both cohorts failed to reject the null hypothesis of the Anderson-Darling test, the F-test for equal variances at a 0.05 significance level was performed to determine whether a Student’s t or Welch’s test [19] should be performed to determine the significance of differences in mean values between cohorts. If distributions from one or more cohorts rejected the null hypothesis of the Anderson-Darling test, the two-sample Kolmogorov-Smirnov test [20] was used to test whether or not samples came from distributions of the same shape. If the distributions failed to reject the null hypothesis of the Kolmogorov-Smirnov test, the Mann-Whitney U test [21] was used to test for significant differences in the median values between cohorts; else, Welch’s test was used to test for significant differences in the mean values between cohorts. All statistical tests were performed in MATLAB. All probability distribution functions (PDFs) are visualized using kernel density estimation (KDE) [22].

#### Classification

Univariate classification for each measurement was assessed with receiver-operating characteristic (ROC) curve analysis of the PDFs of each cohort. The C-statistic is the area under the ROC curve and a C-statistic of 0.5 indicates a random separation, whereas a C-statistic of 1 indicates a perfect separation. Multivariate classification was assessed with Fisher Discriminant Analysis (FDA) [23] and PDFs were calculated on the resulting FDA scores in a similar manner as for the PDFs of the individual measurements.

#### Data extraction

No published study on fatty acid profiles in ASD discloses raw, individual-level data. Therefore, comparison data were extracted from reported figures in [14, 24]. Briefly, images of each figure were saved and masks of individual markers were manually selected. The center of each marker was identified by cross-correlation and the resulting data points were extracted for further analysis.

## Results

### Univariate statistics and classification

**Fig. 1:**
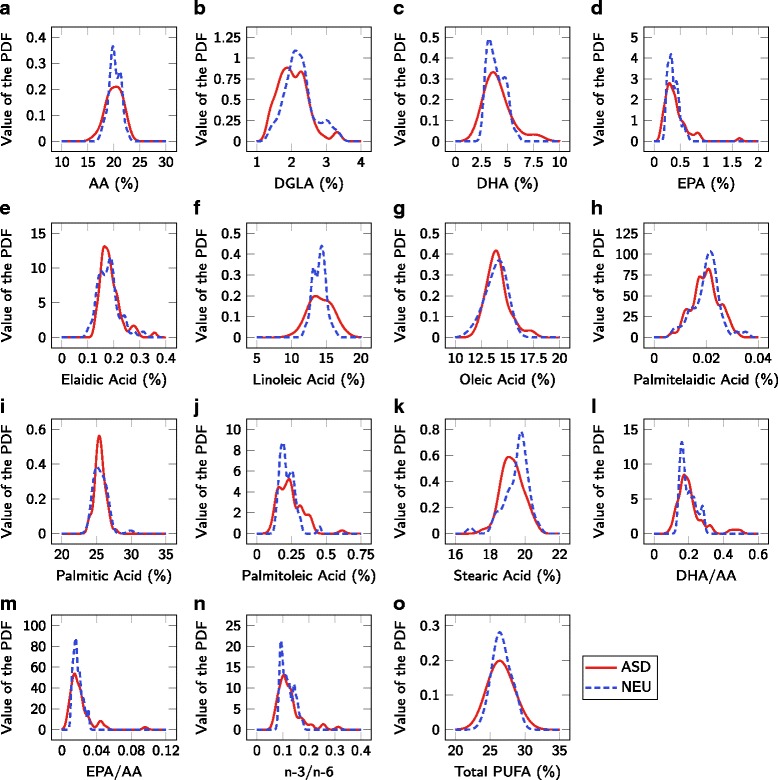
Distributions of fatty acid measurements for ASD and NEU cohorts. Fatty acids investigated are (**a**) AA, (**b**) DGLA, (**c**), DHA, (**d**) EPA, (**e**) elaidic acid, (**f**) linoleic acid, (**g**) oleic acid, (**h**) palmitelaidic acid, (i) palmitic acid, (**j**) palmitoleic acid, (**k**) stearic acid, (**l**) DHA/AA, (**m**) EPA/AA, (**n**) n-3/n-6, and (**o**) Total PUFA. All results are normalized by the concentration of total fatty acids in the sample

Fatty acid measurements were first analyzed for significant differences in mean/median concentration levels (Fig. 1 and Table 3). From Table 3, only DGLA showed significantly different values between the ASD and NEU cohorts (8% lower in ASD, p = 0.03), although stearic acid was marginally significant (2% lower in ASD, p = 0.06). After correction for multiple hypothesis testing, none of these differences are significant. Furthermore, the visualization of the distributions (Fig. 1) illustrate that none of the measurements can be used to classify individual participants due to the significant overlap in the PDFs and this univariate classification is quantified with the C-statistic on the PDFs (Table 3). These results were virtually unaffected by excluding the 8 Asperger’s and 7 PDD-NOS participants. Overall, these results suggest that none of the individual fatty acids measured can be used alone as a diagnostic biomarker for ASD.

**Table 3:** Univariate tests for group mean/median differences between ASD and NEU cohorts

Measurement	Statistical test	Mean/Median values [95% CI]		*p*-value	C-statistic
		ASD	NEU		
AA	Welch’s test	20.05 [17.04, 22.76]	20.16 [18.12, 22.03]	0.68	0.51
DGLA	Student’s t-test	2.045 [1.39, 2.80]	2.226 [1.548, 3.110]	0.03	0.62
DHA	Mann-Whitney U test	3.707 [2.05, 7.11]	3.816 [2.721, 5.347]	1.00	0.50
EPA	Mann-Whitney U test	0.349 [0.158, 0.828]	0.344 [0.225, 0.569]	1.00	0.51
Elaidic acid	Mann-Whitney U test	0.177 [0.136, 0.274]	0.176 [0.120, 0.253]	0.37	0.55
Linoleic acid	Welch’s test	14.18 [11.17, 17.26]	14.00 [12.43. 15.61]	0.45	0.53
Oleic acid	Mann-Whitney U test	13.90 [12.36, 16.31]	13.94 [11.86, 15.55]	0.94	0.52
Palmitelaidic acid	Student’s t-test	0.0198 [0.0110, 0.0286]	0.0201 [0.0105, 0.0282]	0.76	0.52
Palmitic acid	Mann-Whitney U test	25.50 [24.14, 26.87]	25.44 [24.04, 27.15]	0.93	0.50
Palmitoleic acid	Mann-Whitney U test	0.229 [0.124, 0.389]	0.205 [0.0147, 0.306]	0.30	0.56
Stearic acid	Welch’s test^a^	19.27 [18.23, 20.41]	19.51 [18.24, 20.49]	0.06	0.62
DHA/AA	Mann-Whitney U test	0.190 [0.116, 0.350]	0.182 [0.140, 0.280]	0.91	0.51
EPA/AA	Mann-Whitney U test	0.0165 [0.0073, 0.0462]	0.0168 [0.0110, 0.0292]	0.95	0.51
n-3/n-6	Mann-Whitney U test	0.111 [0.0707, 0.224]	0.113 [0.0850, 0.163]	0.94	0.50
Total PUFA	Student’s t-test	26.54 [23.35, 29.90]	26.65 [24.34, 29.11]	0.69	0.52

^a^indicates that Welch’s test was used after the distributions were found to not be of the same shape

Median values are provided if the Mann-Whitney U test was used for comparison; otherwise, mean values are provided. Brackets indicate 95% confidence intervals (CIs) obtained from the estimated PDFs

### Multivariate classification

**Fig. 2:**
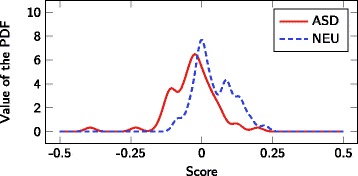
Probability distributions of fitted FDA scores. All fatty acid measurements were included

Multivariate classification using FDA was then used to examine whether combinations of fatty acid measurements could be used to generate a diagnostic biomarker for ASD. All variables presented in Table 3 were included in the FDA analysis. PDFs of the FDA scores are provided in Fig. 2 and this multivariate classifier has a C-statistic of 0.76. Although the multivariate results seem to be an improvement over the univariate classification in Table 3, the multivariate classification has many more variables and a validation strategy would be needed to compare the univariate and multivariate classification. However, because the multivariate classification did not generate sufficient diagnostic accuracy, further validation schemes (see the cross-validatory approach in [25] for an example) were not performed as fitting results will almost always outperform prediction results, i.e., if multivariate classification without cross-validation does not perform well then classification with cross-validation will not result in acceptable results either. Similarly, identifying a subset of input variables to avoid overfitting was unnecessary here as fitting performance of a subset of variables will at best be as good as fitting a classifier using all inputs. Since the performance for all inputs is rather poor, the performance of a subset of inputs for fitting will be even worse and therefore was not investigated.

For the sake of completeness, regression analysis of 12 measures of ASD severity [17] against combinations of fatty acids has been performed using partial least squares and its nonlinear extension kernel partial least squares. The prediction accuracy was generally poor with low R^2^ values even for the best combinations of fatty acids.

### Regression with seafood intake

Using the information provided by the caregiver questionnaire, the red blood cell fatty acid compositions were regressed onto the number of seafood meals per month (Table 4). The distribution of seafood meals per month was similar between the ASD and NEU cohorts. Unsurprisingly, increased seafood consumption was modestly correlated with increased DHA and EPA in ASD, NEU, and ASD+NEU cohorts. Increased seafood consumption was also correlated with decreased stearic acid in the NEU cohort, which may be due in part to the increased concentrations of EPA and DHA. These results support the link [26, 27] between dietary seafood intake and increased levels of EPA and DHA.

**Table 4:** Regression of red blood cell fatty acids onto seafood consumption per month for each listed group

Red blood cell	Correlation coefficient		
Fatty acids	ASD	NEU	ASD + NEU
AA	− 0.204	− 0.107	− 0.183
DGLA	− 0.033	− 0.002	− 0.039
DHA	0.347	0.477	0.374
EPA	0.400	0.320	0.393
Elaidic acid	0.021	0.154	0.062
Linoleic acid	− 0.022	0.120	0.008
Oleic acid	− 0.108	0.017	− 0.066
Palmitelaidic acid	0.135	− 0.061	0.074
Palmitic acid	0.040	− 0.076	− 0.002
Palmitoleic acid	0.117	0.041	0.112
Stearic acid	− 0.173	− 0.498	− 0.263
DHA/AA	0.369	0.477	0.392
EPA/AA	0.391	0.316	0.386
n-3/n-6	0.363	0.466	0.385
Total PUFA	0.150	0.241	0.164

## Discussion

**Fig. 3:**
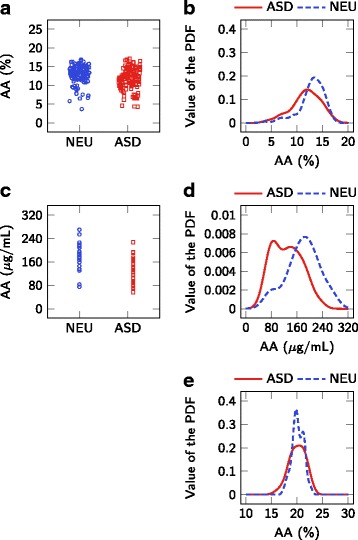
Comparison of distributions of AA. Comparison of (**a**, **b**) the results presented in Brigandi et al. [14], (**c**, **d**) the results presented in Yui et al. [24], and (**e**) the results presented in this work

**Fig. 4:**
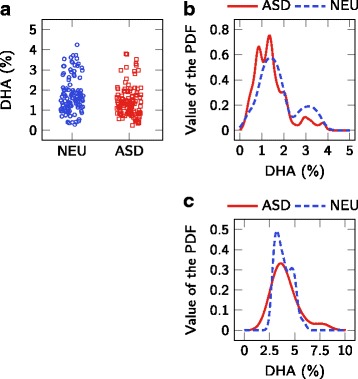
Comparison of distributions of DHA. Comparison of (**a**, **b**) the results presented in Brigandi et al. [14] and (**c**) the results presented in this work

The results presented herein suggest that the measured erythrocyte fatty acids are not predictive of ASD status. A strength of this study is a larger sample size than most other studies, with a control group matched for age and gender. A limitation is the wide age range of groups; a narrower age range, or a younger age range, may find smaller differences. Although these results may seem to contrast those found from some of the previous studies presented in Table 1, most of the apparent discrepancy can be explained by the evaluation of results. Other than [13], previous studies do not evaluate red blood cell fatty acid biomarkers on an individual level, but rather focus on differences in mean metabolite levels over the entire population. This population-level assessment of biomarkers only accounts for differences in the center of the distribution and does not account for the width of the distributions, an effect that is appropriately accounted for in an individual-level assessment. For example, Fig. 3 compares results for AA from this study with those extracted from [14, 24]. Despite the statistically significant lower AA in the ASD population when compared with the NEU population reported in [14, 24], the PDFs indicate that there is only slight separation between these groups at the individual level (C-statistic =0.62 and 0.75, respectively, on the extracted data). The reported values for AA are different between studies due to disparate methods for quantifying fatty acid profiles; however, the data suggests that the AA concentration in erythrocyte membranes is not useful as a biomarker for predicting ASD status. The exact same effect can be seen for DHA measurements in Fig. 4: a statistically significant difference in mean DHA levels does not indicate its usefulness as a biomarker for ASD status. This effect is also seen in the results from this paper, with a statistically significant lower population mean for DGLA (*p*=0.03), but a C-statistic of only 0.62. These results highlight a common challenge in biomarker research where significant differences at the population level should not be equated with significant classification at the individual level.

There are other methods beyond calculation of the C-statistic that can provide similar insights into the biological relevance of the hypothesis under investigation. In particular calculation of effect sizes and CIs usually provide more insight into the underlying biological hypothesis than null hypothesis significance testing and many fields, including clinical trial research, are beginning to move toward these approaches for reporting research findings [28, 29]. In particular, these approaches can include information on the spread of the distributions under investigation, which is usually as, if not more, important than the sample means in biological classification problems. The null hypothesis significance testing conducted in this work is used mainly to illustrate that these methods can lead to inappropriate conclusions that can be rectified by using CIs or the C-statistic to quantify differences between two populations.

Biomarkers represent the “holy grail” of precision medicine [30] in that they quantify changes in single molecules or even entire molecular pathways and quantitatively link clinical outcomes with physiology in health and disease [31]. Despite their promise, translating biomarker research into clinical practice is poor with a less than one percent success rate [32, 33]. Many of these failed biomarkers persist in the literature due to a lack of access to raw data (hence, the reliance on data extraction from published figures in this paper) and a culture that does not credit negative results [31]. Biomarker research in ASD would benefit from improving data access, embracing negative results, and focusing on individual-level classification (with a validation strategy such as cross-validation) [31, 33] to more quickly reach diagnostics and treatments that positively impact those with ASD.

It is important to note that this study did not investigate the therapeutic effects of fatty acid supplementation. Recent meta-analyses reach conflicting conclusions [11, 34, 35], some of which may be attributed to small sample sizes, low doses, and inadequate lengths of supplementation and observational time frames. However, this study indicates that there are no differences between fatty acid levels in ASD and NEU cohorts; therefore, fatty acid therapeutics would need to achieve a different fatty acid profile than the average NEU profile for possible therapeutic benefit.

## Conclusion

The results of this study suggest that fatty acid profiles are similar between individuals with ASD and NEU controls; therefore, fatty acid profiles are not promising biomarkers for classifying ASD and NEU children. A repository of individual-level measurements in biomarker studies for ASD, including those reporting negative results, would greatly help the field iterate toward more promising biomarkers for classifying ASD.

## Additional file

Additional file 1 Individual level fatty acid measurements. Deidentified individual-level data analyzed in this work. (CSV 11 kb)

## References

[1] American Psychiatric Association (2013). Diagnostic and Statistical Manual of Mental Disorders, 5th edn.

[2] Centers for Disease Control. Prevalence of Autism Spectrum Disorders – Autism and Developmental Disabilities Monitoring Network, 14 Sites, United States, 2008. 2012. http://www.cdc.gov/mmwr/preview/mmwrhtml/ss6103a1.htm. Accessed 17 Mar 2016. 22456193

[3] Boyle CA, Boulet S, Schieve LA, Cohen RA, Blumberg SJ, Yeargin-Allsopp M, Visser S, Kogan MD (2011). Trends in the prevalence of developmental disabilities in US children, 1997–2008. Pediatrics.

[4] van Heijst BF, Geurts HM (2015). Quality of life in autism across the lifespan: A meta-analysis. Autism.

[5] Buescher A, Cidav Z, Knapp M, Mandell D (2014). Costs of autism spectrum disorders in the United Kingdom and the United States. JAMA Pediatr.

[6] Di Martino A, Yan CG, Li Q, Denio E, Castellanos FX, Alaerts K, Anderson JS, Assaf M, Bookheimer SY, Dapretto M (2014). The autism brain imaging data exchange: Towards a large-scale evaluation of the intrinsic brain architecture in autism. Mol Psychiatry.

[7] Chen JA, Peñagarikano O, Belgard TG, Swarup V, Geschwind DH (2015). The emerging picture of autism spectrum disorder: Genetics and pathology. Annu Rev Pathol Mech Dis.

[8] de la Torre-Ubieta L, Won H, Stein JL, Geschwind DH (2016). Advancing the understanding of autism disease mechanisms through genetics. Nat Med.

[9] Dinel AL, Rey C, Bonhomme C, Le Ruyet P, Joffre C, Layé S (2016). Dairy fat blend improves brain DHA and neuroplasticity and regulates corticosterone in mice. Prostaglandins Leukot Essent Fat Acids (PLEFA).

[10] Kuratko CN, Salem N (2009). Biomarkers of DHA status. Prostaglandins Leukot Essent Fat Acids.

[11] Mazahery H, Stonehouse W, Delshad M, Kruger MC, Conlon CA, Beck KL, von Hurst PR (2017). Relationship between long chain n-3 polyunsaturated fatty acids and autism spectrum disorder: Systematic review and meta-analysis of case-control and randomised controlled trials. Nutrients.

[12] Sergeant S, Ruczinski I, Ivester P, Lee TC, Morgan TM, Nicklas BJ, Mathias RA, Chilton FH (2016). Impact of methods used to express levels of circulating fatty acids on the degree and direction of associations with blood lipids in humans. Br J Nutr.

[13] El-Ansary AK, Bacha AGB, Al- Ayahdi LY (2011). Plasma fatty acids as diagnostic markers in autistic patients from Saudi Arabia. Lipids Health Dis.

[14] Brigandi SA, Shao H, Qian SY, Shen Y, Wu BL, Kang JX (2015). Autistic children exhibit decreased levels of essential fatty acids in red blood cells. Int J Mol Sci.

[15] Bell JG, Miller D, MacDonald DJ, MacKinlay EE, Dick JR, Cheseldine S, Boyle RM, Graham C, O’Hare AE (2010). The fatty acid compositions of erythrocyte and plasma polar lipids in children with autism, developmental delay or typically developing controls and the effect of fish oil intake. Br J Nutr.

[16] Parletta N, Niyonsenga T, Duff J (2016). Omega-3 and omega-6 polyunsaturated fatty acid levels and correlations with symptoms in children with attention deficit hyperactivity disorder, autistic spectrum disorder and typically developing controls. PLoS ONE.

[17] Adams J, Howsmon DP, Kruger U, Geis E, Gehn E, Fimbres V, Pollard E, Mitchell J, Ingram J, Hellmers R, Quig D, Hahn J (2017). Significant association of urinary toxic metals and autism-related symptoms – A nonlinear statistical analysis with cross validation. PLoS ONE.

[18] Anderson TW, Darling DA (1954). A test of goodness of fit. J Am Stat Assoc.

[19] Welch BL (1947). The generalization of ‘Student’s’ problem when several different population variances are involved. Biometrika.

[20] Massey FJ (1951). The Kolmogorov-Smirnov test for goodness of fit. J Am Stat Assoc.

[21] Mann HB, Whitney DR (1947). On a test of whether one of two random variables is stochastically larger than the other. Ann Math Stat.

[22] Silverman BW (1986). Density Estimation for Statistics and Data Analysis.

[23] Fisher R (1936). The use of multiple measurements in taxonomic problems. Ann Eugenics.

[24] Yui K, Imataka G, Kawasaki Y, Yamada H (2016). Down-regulation of a signaling mediator in association with lowered plasma arachidonic acid levels in individuals with autism spectrum disorders. Neurosci Lett.

[25] Howsmon DP, Kruger U, Melnyk S, James SJ, Hahn J (2017). Classification and adaptive behavior prediction of children with autism spectrum disorder based upon multivariate data analysis of markers of oxidative stress and DNA methylation. PLoS Comput Biol.

[26] Katan MB, Deslypere JP, Birgelen APv, Penders M, Zegwaard M (1997). Kinetics of the incorporation of dietary fatty acids into serum cholesteryl esters, erythrocyte membranes, and adipose tissue: An 18-month controlled study. J Lipid Res.

[27] Harris WS, Pottala JV, Sands SA, Jones PG (2007). Comparison of the effects of fish and fish-oil capsules on the n-3 fatty acid content of blood cells and plasma phospholipids. Am J Clin Nutr.

[28] Nakagawa S, Cuthill IC (2007). Effect size, confidence interval and statistical significance: A practical guide for biologists. Biol Rev.

[29] Cumming G (2014). The new statistics: Why and how. Psychol Sci.

[30] Barker AD, Compton CC, Poste G (2014). The National Biomarker Development Alliance: Accelerating the translation of biomarkers to the clinic. Biomark Med.

[31] Poste G, Compton CC, Barker AD (2015). The national biomarker development alliance: Confronting the poor productivity of biomarker research and development. Expert Rev Mol Diagn.

[32] Poste G (2011). Bring on the biomarkers. Nature.

[33] McPartland JC (2016). Considerations in biomarker development for neurodevelopmental disorders. Curr Opin Neurol.

[34] Horvath A, Łukasik J, Szajewska H. *ω*-3 fatty acid supplementation does not affect autism spectrum disorder in children: A systematic review and meta-analysis. J Nutr. 2017:242354. 10.3945/jn.116.242354. 10.3945/jn.116.242354 28077731

[35] Cheng YS, Tseng PT, Chen YW, Stubbs B, Yang WC, Chen TY, Wu CK, Lin PY (2017). Supplementation of omega 3 fatty acids may improve hyperactivity, lethargy, and stereotypy in children with autism spectrum disorders: A meta-analysis of randomized controlled trials. Neuropsychiatr Dis Treat.

[36] Vancassel S, Durand G, Barthélémy C, Lejeune B, Martineau J, Guilloteau D, Andrès C, Chalon S (2001). Plasma fatty acid levels in autistic children. Prostaglandins Leukot Essent Fat Acids (PLEFA).

[37] Meguid NA, Atta HM, Gouda AS, Khalil RO (2008). Role of polyunsaturated fatty acids in the management of Egyptian children with autism. Clin Biochem.

[38] Pastural l, Ritchie S, Lu Y, Jin W, Kavianpour A, Khine Su-Myat K, Heath D, Wood PL, Fisk M, Goodenowe DB (2009). Novel plasma phospholipid biomarkers of autism: Mitochondrial dysfunction as a putative causative mechanism. Prostaglandins Leukot Essent Fat Acids.

[39] Jory J (2016). Abnormal fatty acids in Canadian children with autism. Nutrition.

